# Exposure of Women With and Without Disabilities to Violence and Discrimination: Evidence from Cross-sectional National Surveys in 29 Middle- and Low-Income Countries

**DOI:** 10.1177/08862605221141868

**Published:** 2022-12-21

**Authors:** Eric Emerson, Gwynnyth Llewellyn

**Affiliations:** 1 University of Sydney, NSW, Australia; 2 Lancaster University, UK

**Keywords:** disability, violence, discrimination, safety, attitudes

## Abstract

There is a paucity of robust nationally representative data from low- and middle-income countries (LMICs) on the prevalence and risk factors associated with exposure of women with/without disability to either discrimination or violence. We undertook secondary analysis of data collected in Round 6 of UNICEF’s Multiple Indicator Cluster Surveys (MICS) involving nationally representative data from 29 countries with a total sample size of 320,426 women aged 18 to 49 years. We estimated: (1) prevalence rates for exposure to discrimination and violence among women with/without disabilities in the previous year in a range of LMICs; (2) the relative risk of exposure when adjusted for demographic and contextual characteristics; (3) the relative risk of exposure associated with specific functional difficulties associated with disabilities; and (4) the association between country-level estimates and national wealth and human development potential. Our results indicated that women with disabilities were approximately twice as likely as women without disabilities to be exposed to violence and discrimination in the past year, and approximately one-third more likely to feel unsafe in either their home or local neighbourhood and to be at greater risk of domestic violence. Risk of exposure was associated with national characteristics (national wealth, human development potential) and within country factors, especially relative household wealth and level of education. These results must be of concern on two counts. First, they attest to the ongoing violation of the human rights of women with disabilities. Second, they point to increased exposure among women with disabilities to several well-documented social determinants of poorer health.

It was estimated in the *World Report on Disability* that 1 billion people have a disability, over 80% of who live in the world’s low- and middle-income countries (LMICs) (World Health Organization and the World Bank, 2011). The *World Report* and the more recent UN flagship *Disability and Development Report* (United Nations Department of Economic and Social Affairs, 2019) both highlighted the increased risk that people with disabilities face in being exposed to discrimination and violence; two violations of human rights that are also well-established social determinants of poorer health (Krieger, 2014; World Health Organization, 2002, 2014, 2019).

The *Disability and Development Report* also drew attention to the intersection between disability and gender concluding that “discrimination is a major cause of exclusion of persons with disabilities and impedes persons with disabilities from pursuing equal participation in society. Some groups of persons with disabilities such as women with disabilities, indigenous persons with disabilities and persons with intellectual and psychosocial disabilities face multiple discrimination and are even more disadvantaged” (p. 198). There is, however, a paucity of robust nationally representative data from LMICs on the extent of inequity in exposure of women with/without disability to either discrimination or violence and on the risk factors associated with exposure. For example, following on from the publication of the *World Report*, the World Health Organization commissioned a systematic review of exposure to violence among adults with disabilities (Hughes et al., 2012). While the resulting paper shed an important light on the extent to which adults with disabilities may be at increased risk of exposure to violence, the evidence base was clearly unrepresentative of the global population. Of the 26 studies included in the review, only seven were based on samples that were likely to be representative of national or state/province populations of adults with disabilities (Casteel et al., 2008; Hall & Innes, 2010; Lin et al., 2009; Martin et al., 2006; Rand & Harrell, 2009; Rurangirwa et al., 2006; Silver et al., 2005). All of these were undertaken in high-income countries.

Since then, at least eleven additional papers have been published using samples that were likely to be representative of national or state/provincial populations (Coston, 2019; Emerson & Roulstone, 2014; Emerson et al., 2016; Hahn et al., 2014; Khalifeh et al., 2013; Krnjacki et al., 2016; Mitra & Mouradian, 2014; Mitra, Mouradian, et al., 2016; Olofsson et al., 2014; Rachele et al., 2020; Valentine et al., 2019). All but one of these, a study of exposure of women to intimate partner violence (IPV) in Uganda (Valentine et al., 2019), were undertaken in high-income countries. In addition to published papers, data from the Eurostat Database from four European upper-middle income countries in 2017 indicated that adults with disability were 3% more likely than non-disabled adults to live in areas with high levels of crime, violence, or vandalism in Bulgaria, 29% more likely in Montenegro, 33% more likely in Serbia, and 68% more likely in North Macedonia (Eurostat Database, 2020).

While numerous studies have investigated the extent to which people with disabilities are exposed to disability discrimination, we are aware of only eight published scientific papers or reports that have investigated the association between disability and exposure to discrimination among adults with and without disability that have used nationally representative samples (Abrams & Houston, 2006; Abrams et al., 2018; Du Mont & Forte, 2016; Emerson et al., 2021; Krnjacki et al., 2018; Namkung & Carr, 2019; Temple, Kelaher, & Williams, 2018; Temple, Kelaher, et al., 2020). Again, all of these were undertaken in high-income countries.

The paucity of nationally representative data on the situation of women with/without disabilities in LMICs is problematic on at least two counts. First, the effective design and implementation of interventions to reduce exposure to discrimination and violence in LMICs requires robust contemporary information on the scale of the problem and risk factors that may indicate the need to target interventions for specific communities or groups of people. Second, monitoring progress toward the elimination of violence and discrimination globally (including LMICs) requires the ongoing collection of nationally representative data that can be disaggregated by disability status. In both cases, it would be grossly inappropriate to assume that data collected, and knowledge generated, in the United States, the United Kingdom, or other high-income countries would necessarily be relevant to the situation of the majority of the world’s population people living in 137 LMICs. The aims of the present study were to begin to redress this inequity in published research by estimating: (1) the prevalence of exposure to discrimination and violence among women with/without disabilities in the previous year in a range of LMICs; (2) relative inequities in exposure when adjusted for demographic factors; (3) the extent to which relative inequities in exposure vary by type of functional difficulty associated with disabilities; (4) risk factors among people with disabilities associated with increased risk of exposure; and (5) the association between country-level estimates of absolute and relative inequity and national wealth and human development potential.

## Method

We undertook a secondary analysis of nationally representative data collected in Round 6 (2017–) of UNICEF’s Multiple Indicator Cluster Surveys (MICS) (Khan & Hancioglu, 2019; UNICEF, 2015). Following the approval by UNICEF, MICS data were downloaded from http://mics.unicef.org/. MICS contains several questionnaire modules. Data used in the present paper were extracted from the household module and the module applied to all women aged 15 to 49 years in the household (Khan & Hancioglu, 2019). All countries used cluster sampling methods to derive samples representative of the national population of women and children. Specific details of the sampling procedures and the procedures for ethical review and approval used in each country are available at http://mics.unicef.org/. At the end of the download period (February 1, 2022), nationally representative survey data containing data on disability status and exposure to either violence or discrimination among women were available for 29 LMICs (14 upper-middle, 10 lower-middle, and 5 low-income countries; see Table 1). MICS has recently developed an optional module on the well-being of men. However, uptake in Round 6 was limited (approximately 50% in the sample of countries included in the present analyses) and the sampling frame used results in markedly small sample sizes for men. For these methodological reasons, and the point made in the introduction about the intersectionality of gender, disability, and violence, we decided to focus solely on women.

**Table 1.** Country-Level Survey Data.

Country	Year of Survey	pcGNI (2018)	Response Rate	Sample Size^a^	Prevalence of Disability (With 95% CI)	Prevalence of Exposure to Violence (With 95% CI)	Prevalence of Exposure to Discrimination (With 95% CI)	Prevalence of (With 95% CI) Higher Risk of IPV	Prevalence of Concern About Safety (With 95% CI)
Upper-middle income									
Costa Rica	2018	$11,590	82.5%	6,902	25.3% (23.6–27.1)	5.8% (4.9–6.7)	25.4% (23.5–27.3)	3.3% (2.8–4.0)	59.1% (56.3–61.9)
Montenegro	2018/19	$8,430	54.9%	2,753	10.3% (6.1–16.9)	2.5% (1.0–6.1)	6.4% (5.0–8.3)	10.9% (5.6–20.0)	22.6% (15.0–32.7)
Dominican Republic	2019	$7,760	97.0%	20,029	14.4% (13.5–15.2)	10.5% (9.8–11.3)	10.8% (10.2–11.5)	2.1% (1.8–2.4)	56.3% (54.9–57.6)
Cuba	2019	$7,480	97.7%	8,401	5.9% (4.9–7.0)	0.7% (0.5–1.1)	2.2% (1.7–2.8)	1.6% (1.2–2.0)	13.4% (11.9–15.0)
Turkmenistan	2019	$6,740	96.0%	6,973	4.9% (4.2–5.6)	0.1% (0.1–0.2)	2.7% (2.3–3.3)	49.2% (46.6–51.8)	24.8% (22.8–27.0)
Guyana	2019/20	$6,290	84.2%	5,290	19.0% (17.3–20.9)	5.9% (4.9–7.2)	14.5% (13.2–15.9)	10.4% (9.1–11.9)	44.9% (41.9–48.0)
Belarus	2019	$5,700	93.4%	5,270	8.2% (7.0–9.4)	0.8% (0.6–1.2)	4.8% (4.1–5.6)	3.9% (3.2–4.7)	35.7% (33.7–37.7)
North Macedonia	2018/19	$5,470	83.8%	4,245	19.3% (12.1–29.5)	n/a	14.1% (11.4–17.2)	12.5% (9.0–17.1)	n/a
Tuvalu	2019/20	$5,430	94.6%	762	14.8% (13.6–16.1)	5.9% (4.7–7.5)	30.1% (23.2–38.1)	44.7% (44.5–44.9)	42.9% (40.0–45.8)
Suriname	2018	$5,210	74.0%	6,261	16.8% (15.9–17.7)	3.3% (2.9–3.8)	8.5% (7.8–9.2)	4.4% (3.9–4.9)	54.8% (53.6–56.0)
Iraq	2018	$5,040	98.2%	26,752	15.6% (14.7–16.6)	1.6% (1.3–2.0)	11.7% (10.2–13.3)	37.5% (35.8–39.3)	50.2% (48.4–52.0)
Georgia	2018	$4,450	75.4%	6,461	21.0% (19.6–22.5)	0.8% (0.5–1.2)	6.5% (5.6–7.5)	n/a	19.5% (17.9–21.3)
Kosovo	2019/20	$4,340	73.6%	5,571	20.1% (18.8–21.4)	n/a	13.3% (12.3–14.5)	30.6% (28.9–32.5)	41.9% (40.1–43.8)
Tonga	2019	$4,300	90.3%	2,493	8.9% (6.8–11.6)	1.6% (0.9–2.8)	24.2% (20.1–28.9)	40.2% (36.2–44.4)	20.7% (17.8–24.0)
Lower-middle income									
Palestine	2019/20	$4,190	92.9%	9,794	8.1% (7.3–9.1)	3.4% (2.8–4.0)	12.8% (11.7–13.9)	15.5% (14.2–17.0)	33.7% (31.8–35.6)
Samoa	2019/20	$4,020	88.9%	3,695	7.4% (6.3–8.8)	1.0% (0.7–1.5)	17.2% (15.2–19.4)	37.1% (33.8–40.4)	20.2% (18.0–22.7)
Mongolia	2018	$3,660	90.4%	9,872	22.2% (20.7–23.7)	5.9% (5.2–6.7)	10.3% (9.1–11.6)	9.3% (8.3–10.4)	49.3% (46.9–51.6)
Tunisia	2018	$3,500	93.8%	9,788	25.9% (25.0–26.8)	3.0% (2.7–3.4)	15.8% (15.1–16.5)	15.1% (14.4–15.8)	27.1% (26.2–28.0)
Kiribati	2018/19	$3,140	96.7%	3,806	20.1% (18.2–22.3)	4.6% (3.9–5.5)	31.0% (29.1–32.9)	71.4% (68.4–74.2)	50.4% (47.4–53.3)
Honduras	2019	$2,320	86.0%	17,137	23.7% (22.8–24.6)	5.4% (4.9–5.9)	11.5% (10.9–12.2)	5.9% (5.5–6.4)	40.6% (39.2–41.9)
Zimbabwe	2018/19	$1,790	92.8%	8,888	11.5% (10.8–12.2)	6.0% (5.4–6.6)	20.3% (19.0–21.5)	n/a	53.1% (51.3–54.9)
Bangladesh	2019	$1,750	93.1%	57,699	10.5% (10.2–10.9)	4.0% (3.8–4.2)	9.9% (9.6–10.3)	26.6% (26.1–27.1)	24.6% (24.1–25.1)
Lesotho	2018	$1,390	86.0%	5,630	12.2% (11.1–13.5)	6.2% (5.5–7.1)	21.3% (19.8–23.0)	22.6% (21.0–24.4)	76.4% (74.8–78.0)
Kyrgyz Republic	2018	$1,220	97.2%	5,164	12.4% (11.5–13.3)	2.6% (2.2–3.1)	7.9% (7.2–8.7)	30.7% (29.5–32.0)	54.0% (52.6–55.4)
Low income									
Chad	2019	$680	98.6%	19,266	16.7% (15.4–18.0)	5.1% (4.4–6.0)	17.3% (15.9–18.8)	78.6% (77.0–80.1)	39.2% (37.1–41.3)
Madagascar	2018	$510	89.1%	14,872	21.5% (20.5–22.6)	5.2% (4.6–5.8)	20.7% (19.5–21.9)	40.9% (39.3–42.6)	56.1% (54.0–58.2)
DR Congo	2017/18	$490	99.6%	18,978	12.5% (11.0–14.1)	5.9% (4.9–7.1)	23.2% (20.7–26.0)	62.5% (58.4–66.4)	42.6% (38.8–46.4)
Central African Republic	2018/19	$490	92.2%	8,122	30.2% (28.6–32.0)	6.4% (5.5–7.5)	38.1% (36.0–40.2)	66.2% (63.8–68.5)	56.9% (53.9–59.8)
Malawi	2019/20	$350	94.6%	21,124	11.5% (10.8–12.3)	5.8% (5.3–6.2)	21.1% (20.2–22.0)	18.1% (17.2–19.0)	65.5% (64.2–66.9)

*Note*. IPV = intimate partner violence.

a Sample sizes are unweighted and for women aged 18 to 49 years with valid disability data.

### Disabilities

In Round 6 of MICS the Washington Group Short Set of Questions on Disability (WGSS) were introduced to identify women with disability aged 18 to 49 years. Developed by the Washington Group on Disability Statistics (WGDS: http://www.washingtongroup-disability.com/), the module is based on informant report of difficulties in six different functional domains (seeing, hearing, walking, remembering/concentrating, self-care, and communicating). Four response options were available for each domain (“*no difficulty*,” “*yes—some difficulty*,” “*yes—a lot of difficulty*,” and “*cannot do at all*”). Disability is defined by the WGDS as having “a lot of difficulty” or “cannot do at all” in one or more domains. We used the WGDS criteria for identifying respondents with “more severe” disability. Given that concern has been expressed about the under identification of disability by the WGDS (Bourke et al., 2021; Office for National Statistics, 2019; Sabariego et al., 2015), we also used the procedure outlined by Bourke and colleagues to identify respondents with “less severe” disability if they were not identified as having “more severe” disability, but did report “some difficulty” in two or more functional domains. Disability data were missing for <0.1% of respondents.

### Violence

Two sets of questions addressed exposure to violence in the previous year with the following preamble: “*Now I would like to ask you some questions about crimes in which you personally were the victim. Let me assure you again that your answers are completely confidential and will not be told to anyone.’*

1. “*In the last three years, that is since (month of interview) (year of interview minus 3), has anyone taken or tried taking something from you, by using force or threatening to use force? Did this last happen during the last 12 months, that is, since* (*month of interview*) (*year of interview minus 1*)?”
2. “*Apart from the incident(s) just covered, have you in the last three years, that is since (month of interview) (year of interview minus 3), been physically attacked? Did this last happen during the last 12 months, that is, since* (*month of interview*) (*year of interview minus 1*)?”

Responses were recoded into one binary variable; had been exposed to violence (assault or robbery) in the last 1 year versus not exposed. Violence data were not collected in two countries (Kosovo and North Macedonia) and were missing for 0.4% of respondents with valid disability data in the remaining countries.

### Risk of IPV

A direct exposure to IPV question is not included in MICS. However, evidence indicates that: (1) cultural attitudes toward IPV have a significant impact on violence levels; and (2) at an individual level, women who agree that IPV involving hitting or beating in any circumstance justified are more likely to be victims of IPV (Abramsky et al., 2011; Cools & Kotsadam, 2017; Heise & Fulu, 2014). For example, Abramsky et al. (2011) reported that women’s belief that in any circumstances IPV was in some manner justifiable was associated with an increased risk of actual exposure to IPV by 40% in Namibia, 60% in Ethiopia, 70% in Japan, and 100% in Bangladesh and Brazil. As a result, we used belief in the justifiability of IPV as a proxy indicator for increased risk of IPV. Data were collected from women on their beliefs in the justifiability of IPV by the following question; “*Sometimes a husband/partner is annoyed or angered by things that his wife does. In your opinion, is a husband/partner justified in hitting or beating his wife in the following situations: [A] If she goes out without telling him? [B] If she neglects the children? [C] If she argues with him? [D] If she refuses to have sex with him? [E] If she burns the food?*” These were recoded into a binary variable of the justifiability of IPV (yes to any of the five scenarios vs. no to all scenarios), as a proxy indicator for increased risk of exposure to IPV. These data were not collected in two countries (Zimbabwe and Georgia) and were missing for 0.5% of respondents with valid disability data in the remaining countries.

### Safety

Two questions were asked of respondents about their perceived safety.

1. “*How safe do you feel walking alone in your neighborhood after dark?*”
2. “*How safe do you feel when you are at home alone after dark?*”

Response options for both questions were: (1) very safe; (2) safe; (3) unsafe; (4) very unsafe; (7) never do this. We recoded responses into a single binary variable; had been exposed to unsafe environment (unsafe/very unsafe neighborhood or home) versus not exposed. Safety data were not collected in one country (North Macedonia) and were missing for <0.1% of respondents with valid disability data in the remaining countries.

### Discrimination

Exposure to discrimination was based on responses to a single question: “In the past 12 months, have you personally felt discriminated against or harassed on the basis of the following grounds? (1) Ethnic or immigration origin? (2) Sex? (3) Sexual orientation? (4) Age? (5) Religion or belief? (6) Disability? (7) For any other reason?” Responses to this variable were used to derive a binary variable; exposed to any form of discrimination in the past 12 months versus not exposed. Discrimination data were collected in all countries and were missing for 0.5% of respondents with valid disability data.

### Country Characteristics

We used World Bank 2018 country classification as upper-middle income, lower-middle income, and low income (World Bank, 2021c). These classifications are based on per capita Gross National Income adjusted for purchasing power parity (pcGNI; expressed as current US$ rates) using the World Bank’s Atlas Method. We downloaded 2018 Atlas Method pcGNI from the World Bank website between May 2020 and December 2021 (World Bank, 2021a, 2021b). Data were available for all countries.

In addition for each country we downloaded the country-level 2018 estimate for human development potential, the Human Development Index (HDI): The composite HDI integrates three dimensions of human development: life expectancy at birth; mean years of schooling and expected years of schooling; and gross national income per capita (Anand & Sen, 1994; United Nations Development Programme, 2016). The HDI is the geometric mean of normalized indices for each of the three dimensions. HDI data for 2018 were taken from the 2019 Human Development Report (United Nations Development Programme, 2019). HDI data were available for all but one country (Tuvalu).

### Household Wealth

MICS data include a within-country wealth index for each household. To construct the wealth index, principal components analysis is performed by using information on the ownership of consumer goods, dwelling characteristics, water and sanitation, and other characteristics that are related to the household’s wealth, to generate weights for each item. Each household is assigned a wealth score based on the assets owned by that household weighted by principle component factor scores. The wealth index is assumed to capture underlying long-term wealth through information on household assets (Howe et al., 2012; Poirier et al., 2020; Rutstein, 2008; Rutstein & Johnson, 2004). Data were collected in all countries with no missing data.

### Highest Level of Education

The highest level of education of each woman in the household was recorded using country-specific categories. We recoded these data into a three-category measure: (1) no or pre-primary education; (2) primary education; (3) secondary or higher-level education. Data were collected in all countries and were missing for <0.1% of respondents with valid disability data.

### Urban/Rural Location

Data were released with a within-country defined binary indicator of urban/rural location for each household. Data were missing for one country (Belarus). No data were missing in other countries.

### Approach to Analysis

In the first stage of analysis, we used simple bivariate descriptive statistics to derive country-level and pooled estimates (with 95% confidence intervals) of the prevalence of disability, exposure to violence, risk of IPV, unsafe environments, and discrimination. In the second stage of analysis, we used mixed effects multilevel multivariate Poisson regression to estimate the independent strength of association (adjusted prevalence rate ratios [APRRs]) between selected demographics and contextual factors (age, household wealth, highest level of education attained, and urban/rural location) and the overall risk of experiencing disability, and being exposed to violence, higher risk of IPV, unsafe environments, and discrimination.

In the third stage of analysis, we used simple bivariate descriptive statistics to estimate country-level prevalence rates for women with/without disability being exposed to violence, higher risk of IPV, unsafe environments, and discrimination. We also used and Poisson regression to estimate prevalence rate ratios for women with disabilities being exposed to these adversities adjusted for between-group differences in age (women without disabilities being the reference group). We also used two different methods to generate pooled results across countries: (1) mixed effects multilevel modelling with random effects specified to allow the intercept and slope for the association between disability and the outcome to vary across countries; (2) restricted maximum likelihood meta-analysis.

In the fourth stage of analysis, we used mixed effects multilevel modelling to investigate the extent to which risk of exposure to violence, discrimination, risk of IPV, and perceptions of safety varied with the type of functional difficulty associated with the respondent’s disabilities. Again, random effects were included to allow the intercept and slope for the association between impairment type and outcome to vary across countries.

In the fifth stage of analysis, we used mixed effects multilevel modelling to investigate the extent to which risk of exposure to violence, discrimination, risk of IPV, and perceptions of safety among women with disabilities varied by within country demographic characteristics. These analyses were also stratified by country economic classification to estimate the stability of results between upper-middle, lower-middle, and low-income countries.

In the final stage of analysis, we used Spearman’s non-parametric correlation to investigate the strength of association between country wealth, estimates of human development potential (HDI), and country-level estimates generated by the above analyses. In addition to the data reported in Tables 1 and 3, we also computed the absolute inequality between women with/without disabilities for our main outcome measures. Absolute inequality was defined as the difference in the number of percentage points between women with/without disabilities for each indicator. Given the limitations of null hypothesis significance testing when applied to relatively small samples (Wasserstein & Lazar, 2016), in this case 29 countries, as well as reporting statistically significant associations, we also draw attention to associations that while not meeting the alpha level of *p* < .05 are commonly considered to be indicative of medium or larger effect sizes; *r* ≥ .3 (Cohen, 1992).

All analyses were undertaken using Stata 16.1 using svy facility to take account of the clustering of observations by country and within country sampling clusters. Mixed effects multilevel modelling of within-country associations was undertaken using the mepoisson commands to generate APRRs. UNICEF’s country-specific women-level weights were used to take account of biases in sampling frames and household and individual level non-response. Given the small amount of missing data, complete case analyses were undertaken.

## Results

Information (including sample sizes, response rates and the prevalence of disability, exposure to violence, discrimination, risk of IPV, and unsafe environments) for the 29 surveys is presented in Table 1.

### Prevalence and Predictors of Disability

Overall, 15.2% (95% CI [14.4–16.0]) of women aged 18 to 49 years were identified as having a disability (9.8% [9.2–10.5] with less severe disability and 5.3% [5.1–5.5] with more severe disability). The most frequently reported functional difficulties were remembering (10.5% [9.8–11.3]), seeing (9.1% [8.8–9.4]), walking (8.5% [7.7–9.4]), hearing (3.5% [3.4–3.6]), communicating (2.3% [2.2–2.4]), and self-care (1.6% [1.5–1.8]). Multilevel multivariate modelling indicated that increased risk of disability was independently associated with older age, lower household wealth, lower education, and urban location (Table 2).

**Table 2.** Predictors of Risk of Disability, Violence, Discrimination, Acceptability of Partner to Wife Violence, and Low Safety.

	Disability (29 Countries, *n* = 321,959)	Violence (27 Countries, *n* = 310,978)	Discrimination (29 Countries, *n* = 320,426)	Higher Risk of IPV (27 Countries, *n* = 305,038)	Concern About Safety (28 Countries, *n* = 317,617)
Age					
18–19	1.00 (reference)	1.00 (reference)	1.00 (reference)	1.00 (reference)	1.00 (reference)
20–24	0.98 (0.91–1.07)	1.21*** (1.12–1.31)	0.98 (0.95–1.02)	0.98 (0.94–1.03)	0.96*** (0.94–0.98)
25–29	1.11 (0.98–1.27)	1.18* (1.03–1.36)	0.96* (0.92–0.99)	0.99 (0.92–1.07)	0.90*** (0.88–0.92)
30–34	1.31** (1.10–1.56)	1.12 (0.97–1.29)	0.97 (0.93–1.01)	0.99 (0.90–1.09)	0.86*** (0.84–0.88)
35–39	1.76*** (1.38–2.23)	1.04 (0.94–1.16)	0.98 (0.94–1.02)	0.99 (0.89–1.10)	0.82*** (0.80–0.84)
40–44	2.44*** (1.86–3.21)	0.97 (0.86–1.11)	0.95* (0.92–0.99)	0.98 (0.89–1.08)	0.79*** (0.78–0.81)
45–49	3.23*** (2.48–4.20)	0.85* (0.75–0.97)	0.96 (0.92–1.00)	0.97 (0.88–1.07)	0.78*** (0.76–0.80)
Household wealth					
Quintile 1 (poorest)	1.33*** (1.20–1.48)	1.39 (0.99–1.94)	1.24*** (1.20–1.29)	1.41** (1.11–1.78)	1.11*** (1.09–1.14)
Quintile 2	1.24*** (1.11–1.38)	1.28 (0.99–1.67)	1.16*** (1.12–1.20)	1.32* (1.07–1.63)	1.11*** (1.09–1.13)
Quintile 3	1.18*** (1.09–1.28)	1.11 (0.90–1.38)	1.12*** (1.08–1.15)	1.23** (1.06–1.42)	1.09*** (1.07–1.11)
Quintile 4	1.13*** (1.06–1.20)	1.09 (0.91–1.30)	1.08*** (1.04–1.12)	1.17** (1.05–1.30)	1.08*** (1.06–1.10)
Quintile 5 (wealthiest)	1.00 (reference)	1.00 (reference)	1.00 (reference)	1.00 (reference)	1.00 (reference)
Education					
None/pre-primary	1.32*** (1.17–1.48)	0.95 (0.84–1.08)	1.01 (0.98–1.05)	1.36*** (1.18–1.56)	0.93*** (0.91–0.95)
Primary	1.30*** (1.20–1.40)	1.01 (0.91–1.12)	1.03* (1.01–1.06)	1.32*** (1.18–1.49)	0.98* (0.97–1.00)
Secondary or higher	1.00 (reference)	1.00 (reference)	1.00 (reference)	1.00 (reference)	1.00 (reference)
Location					
Rural	1.00 (reference)	1.00 (reference)	1.00 (reference)	1.00 (reference)	1.00 (reference)
Urban	1.08* (1.01–1.14)	1.40*** (1.19–1.64)	1.11*** (1.09–1.14)	0.93 (0.86–1.01)	1.11*** (1.09–1.12)

*Note*. Belarus excluded from analyses as urban/rural data not collected.

* *p* < .05. **p < .01. ***p < .001.

### Prevalence and Predictors of Exposure to Violence, Discrimination, Risk of IPV, and Perceptions of Safety

Overall exposure rates among women aged 18 to 49 years were 4.5% (95% CI [4.3–4.7]) for violence, 14.4% (95% CI [14.1–15.0]) for discrimination, 28.9% (95% CI [27.0–30.8]) for risk of IPV, and 42.0% (95% CI [41.5–42.4]) for low safety. Multilevel multivariate modelling indicated that increased risk of exposure to all four outcomes was independently associated with lower household wealth (Table 2). In addition, increased exposure to violence was associated with younger age and urban location, increased exposure to discrimination was associated with lower educational level, younger age and urban location, increased risk of IPV was associated with lower educational level, and increased risk of concern about safety was associated with higher educational level.

### Disability and Exposure to Violence, Discrimination, Risk of IPV, and Perceptions of Safety

The prevalence of exposure of women with/without disability to violence, discrimination, higher risk of IPV, and increased risk of concern about safety and in each country is presented in Table 3 along with age-APPRs for women with disability (women without disability being the reference group). Exposure to violence was higher among women with disabilities in all 27 countries, significantly so in 24. Exposure to discrimination was significantly higher among women with disabilities in all 29 countries. Risk of IPV was higher among women with disabilities in all 27 countries, significantly so in 21. Increased risk of concern about safety was higher among women with disabilities in all 28 countries, significantly so in 27.

**Table 3.** Prevalence of Exposure to Violence Among Women With/Without Disabilities With Age-APRR for Women With Disabilities.

Exposure	Violence			Discrimination			Higher Risk of IPV			Concern About Safety		
Disability	Yes	No	APRR	Yes	No	APRR	Yes	No	APRR	Yes	No	APRR
Costa Rica	7.6% (5.9–9.7)	5.2% (4.3–6.2)	1.57** (1.16–2.13)	33.3% (29.9–36.9)	22.7% (20.5–25.1)	1.48*** (1.26–1.73)	4.9% (3.5–6.8)	2.8% (2.2–3.5)	1.69* (1.13–2.55)	68.0% (64.3–71.6)	56.1% (53.0–59.2)	1.21*** (1.14–1.29)
Montenegro	10.5% (3.3–28.6)	1.8% (0.8–4.4)	7.06*** (3.37–14.81)	12.6% (8.4–18.4)	5.7% (4.5–7.3)	2.29*** ,(1.54–3.41)	19.6% (10.7–33.2)	9.9% (5.1–18.1)	2.07*** (1.45–2.96)	47.0% (38.9–55.3)	19.9% (13.2–28.8)	2.77*** (2.04–3.77)
Dominican Republic	14.8% (12.9–16.8)	9.8% (9.0–10.6)	1.59*** (1.36–1.85)	17.8% (15.8–19.9)	9.7% (9.0–10.4)	1.92*** (1.67–2.23)	2.9% (2.1–4.0)	2.0% (1.7–2.3)	1.52* (1.09–2.12)	62.9% (60.3–65.5)	55.1% (53.7–56.6)	1.13*** (1.08–1.19)
Cuba	1.5% (0.6–3.7)	0.7% (0.4–1.1)	2.08 (0.63–6.88)	9.8% (5.3–17.2)	1.7% (1.3–2.2)	5.92*** (2.92–12.01)	1.8% (0.8–3.9)	1.6% (1.2–2.0)	1.12 (0.46–2.71)	24.3% (17.7–32.5)	12.7% (11.3–14.3)	1.97*** (1.46–2.66)
Turkmenistan	0.0% (0.0–0.7)	0.1% (0.0–0.2)	1.55 (0.16–15.26)	7.8% (4.9–12.2)	2.5% (2.0–3.0)	2.88*** (1.72–4.83)	46.5% (39.8–53.2)	49.3% (46.7–52.0)	1.02 (0.89–1.18)	33.7% (27.8–40.2)	24.4% (22.3–26.6)	2.11*** (1.79–2.49)
Guyana	8.5% (4.2–11.2)	5.3% (4.2–6.7)	1.63* (1.10–2.39)	24.6% (20.9–28.8)	12.1% (10.7–13.7)	2.13*** (1.73–2.62)	15.5% (12.3–19.2)	9.3% (7.9–10.8)	1.63*** (1.25–2.14)	51.5% (46.7–56.2)	43.4% (40.1–46.7)	1.20** (1.08–1.33)
Belarus	3.2% (1.7–6.2)	0.6% (0.4–0.9)	6.46*** (3.03–13.77)	9.6% (6.4–14.3)	4.4% (3.7–5.2)	2.22** (1.41–3.49)	6.8% (4.3–10.5)	3.6% (3.0–4.5)	1.72* (1.05–2.84)	40.5% (33.8–47.5)	35.2% (33.3–37.2)	1.20* (1.01–1.42)
North Macedonia	Data not collected						20.9% (15.9–27.1)	12.4% (10.5–14.6)	2.58*** (1.95–3.41)	Data not collected		
Tuvalu	7.7% (4.2–13.7)	5.6% (3.7–8.5)	1.73 (0.83–3.60)	48.0% (40.2–56.0)	27.0% (20.4–34.8)	1.93*** (1.41–2.63)	45.8% (31.4–60.9)	44.5% (41.7–47.3)	1.01 (0.74–1.37)	54.9% (50.0–59.7)	40.8% (38.4–43.3)	1.43* (1.08–1.89)
Suriname	3.9% (2.9–5.3)	2.6% (2.2–3.0)	1.59* (1.12–2.26)	13.2% (11.2–15.4)	5.5% (4.9–6.1)	2.42*** (1.97–2.98)	7.6% (6.1–9.4)	3.8% (3.3–4.3)	2.10*** (1.61–2.73)	55.2% (52.1–58.2)	50.2% (48.8–51.6)	1.11* (1.02–1.22)
Iraq	2.4% (1.7–3.3)	1.4% (1.1–1.8)	1.82** (1.24–2.68)	17.8% (15.3–21.0)	10.5% (9.2–12.1)	1.86*** (1.61–2.15)	48.4% (43.8–53.1)	35.5% (33.5–53.1)	1.32*** (1.17–1.48)	52.9% (49.7–56.2)	49.7% (47.7–51.7)	1.22*** (1.15–1.29)
Georgia	1.5% (0.8–2.7)	0.6% (0.4–1.1)	3.00** (1.46–6.15)	9.7% (7.4–12.6)	5.6% (4.7–6.7)	1.99*** (1.43–2.76)	Data Not Collected			26.0% (22.5–29.8)	17.8% (16.1–19.7)	1.65*** (1.39–1.94)
Kosovo	Data not collected						20.5% (17.9–23.4)	11.6% (10.5–12.7)	2.23*** (1.90–2.63)	50.5% (47.1–53.9)	39.8% (37.8–41.8)	1.35*** (1.24–1.47)
Tonga	4.0% (1.6–9.5)	1.3% (0.7–2.7)	3.12 (0.87–11.24)	48.9% (41.2–56.6)	21.8% (18.0–26.2)	2.37*** (1.94–2.90)	49.9% (40.5–59.2)	39.2% (35.4–43.2)	1.26* (1.06–1.50)	27.7% (20.8–35.8)	20.0% (17.1–23.3)	1.45** (1.13–1.44)
Palestine	8.6% (6.4–11.6)	2.9% (2.4–3.5)	3.33*** (2.39–4.63)	21.9% (18.5–25.7)	12.0% (10.9–13.1)	1.88*** (1.55–2.28)	26.2% (22.1–30.7)	14.6% (13.3–16.0)	1.87*** (1.58–2.21)	35.3% (31.0–39.7)	33.5% (31.6–35.5)	1.27*** (1.13–1.44)
Samoa	2.7% (1.3–5.4)	0.9% (0.6–1.3)	2.97** (1.35–6.55)	36.2% (29.9–43.1)	15.6% (13.7–17.7)	2.32*** (1.91–2.81)	39.1% (30.7–48.2)	36.9% (33.6–40.3)	1.04 (0.83–1.30)	24.7% (19.4–31.0)	19.9% (17.6–22.4)	1.38** (1.10–1.73)
Mongolia	10.5% (8.7–12.7)	4.5% (3.9–5.3)	2.67*** (2.12–3.37)	17.3% (14.7–20.2)	8.3% (7.0–9.7)	2.26*** (1.82–2.80)	12.5% (10.6–14.8)	8.3% (7.3–9.4)	1.50*** (1.25–1.80)	60.2% (56.3–63.9)	46.2% (43.7–48.6)	1.39*** (1.30–1.50)
Tunisia	4.0% (3.3–4.8)	2.3% (1.9–2.6)	2.10*** (1.62–2.71)	25.1% (23.5–26.9)	13.2% (12.4–13.9)	1.95*** (1.76–2.16)	25.2% (23.6–27.0)	12.6% (11.9–13.4)	1.92*** (1.73–2.13)	36.7% (34.8–38.6)	23.1% (22.1–24.0)	1.68*** (1.55–1.83)
Kiribati	6.4% (4.8–8.6)	4.2% (3.4–5.0)	1.89*** (1.35–2.64)	46.2% (42.0–50.4)	27.1% (25.3–29.1)	1.75*** (1.55–1.96)	73.1% (68.6–77.1)	71.0% (67.7–74.0)	1.02 (0.96–1.08)	57.8% (53.7–61.7)	58.5% (45.5–51.6)	1.25*** (1.17–1.34)
Honduras	7.7% (6.7–8.9)	4.6% (4.2–5.2)	1.85*** (1.57–2.19)	20.5% (10.9–22.0)	8.8% (8.2–9.4)	2.34*** (2.10–2.61)	7.4% (6.5–8.5)	5.5% (5.0–6.0)	1.27** (1.09–1.50)	47.4% (45.2–49.5)	38.4% (37.1–39.8)	1.24*** (1.18–1.30)
Zimbabwe	8.7% (6.9–10.9)	5.6% (5.1–6.3)	1.69*** (1.31–2.17)	34.6% (31.0–38.4)	18.4% (17.2–19.6)	1.85*** (1.65–2.08)	Data Not Collected			56.8% (54.4–60.2)	52.6% (50.7–54.5)	1.18*** (1.11–1.26)
Bangladesh	7.3% (6.6–8.0)	3.6% (3.4–3.8)	2.30*** (2.03–2.60)	14.4% (13.5–15.4)	9.4% (9.1–9.7)	1.85*** (1.71–2.00)	34.6% (33.2–35.9)	25.6% (25.1–26.1)	1.25*** (1.19–1.30)	27.9% (26.6–29.3)	24.2% (23.7–24.8)	1.48*** (1.41–1.57)
Lesotho	9.9% (7.7–12.6)	5.7% (5.0–6.6)	1.79*** (1.36–2.37)	34.6% (30.2–39.1)	19.5% (17.9–21.1)	1.77*** (1.53–2.06)	25.1% (21.6–28.9)	22.3% (20.5–24.2)	1.17 (0.99–1.38)	78.4% (74.4–81.9)	76.2% (74.4–77.8)	1.04 (0.98–1.09)
Kyrgyz Republic	3.9% (2.7–5.7)	2.2% (1.8–2.7)	1.96** (1.25–3.08)	13.2% (10.8–16.0)	5.8% (5.2–6.6)	2.48*** (1.92–3.19)	42.4% (38.7–46.2)	27.0% (25.7–28.3)	1.62*** (1.42–1.86)	56.9% (53.1–60.6)	53.9% (52.4–55.3)	1.13* (1.01–1.27)
Chad	8.1% (6.7–9.8)	4.5% (3.8–5.4)	1.82*** (1.46–2.26)	26.5% (23.2–30.0)	15.4% (14.1–16.9)	1.73*** (1.51–1.98)	81.7% (79.0–84.1)	78.0% (76.3–79.5)	1.05** (1.02–1.09)	46.7% (42.9–50.6)	37.7% (35.6–39.9)	1.26*** (1.15–1.37)
Madagascar	7.9% (6.5–9.5)	4.4% (3.9–5.0)	1.93*** (1.58–2.36)	33.0% (30.4–35.7)	17.3% (16.2–18.5)	1.95*** (1.78–2.14)	47.2% (44.6–49.8)	39.2% (37.4–41.0)	1.23*** (1.15–1.31)	66.3% (63.6–68.8)	53.3% (51.0–55.6)	1.27*** (1.20–1.33)
DR Congo	11.6% (9.1–14.8)	5.1% (4.2–6.3)	2.37*** (1.83–3.08)	42.4% (37.1–47.8)	20.5% (18.2–23.1)	2.12*** (1.84–2.45)	72.0% (67.8–76.0)	61.2% (56.8–65.3)	1.20*** (1.11–1.28)	51.1% (46.2–56.0)	41.1% (37.4–45.4)	1.27*** (1.15–1.41)
Central African Republic	8.3% (6.7–10.2)	5.6% (4.6–6.8)	1.45** (1.14–1.84)	51.0% (47.9–54.2)	32.5% (30.4–34.7)	1.61*** (1.49–1.74)	69.6% (66.7–72.4)	64.7% (62.1–67.2)	1.09*** (1.04–1.13)	62.3% (58.7–65.9)	54.5% (51.2–57.8)	1.15*** (1.08–1.22)
Malawi	10.1% (8.7–11.7)	5.2% (4.7–5.7)	1.95*** (1.65–2.31)	34.4% (32.1–36.8)	19.3% (18.4–20.3)	1.74*** (1.61–1.89)	19.8% (17.7–22.0)	17.9% (16.9–18.8)	1.17** (1.04–1.32)	68.6% (65.3–71.6)	65.1% (63.7–66.6)	1.06** (1.02–1.11)

*Note*. IPV = intimate partner violence; LMIC = low- and middle-income countries.

* *p* < .05. **p < .01. ***p < .001.

Aggregating data across countries using mixed effects multilevel modelling estimated that women with disabilities were 88% more likely to be exposed to discrimination (APRR = 1.88 [1.83–1.93], *p* < .001), 85% more likely to be exposed to violence (APRR = 1.85 [1.70–2.01], *p* < .001), 30% more likely to be at higher risk of IPV (APRR = 1.30 [1.20–1.42], *p* < .001) and 26% more likely to report feeling unsafe (APRR = 1.26 [1.23–1.28], *p* < .001). Aggregating data across countries using meta-analysis estimated that women with disabilities were twice as likely than women without disabilities to be exposed to violence (APRR = 2.02 [1.83–2.23], *p* < .001, *I*^2^ = 64.0%) and discrimination (APRR = 1.97 [1.87–2.08], *p* < .001, *I*^2^ = 69.8%), and one-third more likely to be at higher risk of IPV (APRR = 1.37 [1.25–1.51], *p* < .001, *I*^2^ = 95.8%) and to report feeling unsafe (APRR = 1.32 [1.23–1.42], *p* < 0.001, *I*^2^ = 95.3%). Applying these estimates to the estimated prevalence of disability in LMICs suggests that approximately 25% of women who have been victims of violence or victims of discrimination over the past year were women with disabilities. The results of repeating the mixed effects multilevel modelling for women with more/less severe disability are presented in Table 4. For violence, lack of safety and discrimination, risk was greater for women with more severe disabilities than women with less severe disabilities.

**Table 4.** APRRs (Adjusted for Age) of Women with Disabilities Being Exposed to Violence, Discrimination, Higher Risk of IPV and Unsafe Environments.

	Violence	Discrimination	Higher Risk of IPV	Concern About Safety
No disability	1.00 (reference)	1.00 (reference)	1.00 (reference)	1.00 (reference)
Less severe disability	1.86*** (1.68–2.05)	1.91*** (1.83–2.00)	1.31*** (1.17–1.47)	1.29*** (1.24–1.35)
More severe disability	2.22*** (1.87–2.63)	2.19*** (2.02–2.37)	1.30*** (1.13–1.50)	1.38*** (1.27–1.50)

*Note. Model 1* adjusted for women’s age. *Model 2* adjusted for women’s age, relative household wealth and level of education. APRR = adjusted prevalence rate ratio; IPV = intimate partner violence; LMIC = low- and middle-income countries.

*** *p* < .001.

Disaggregating the discrimination data by form of discrimination indicated that women with disabilities were: nearly three times more likely to have faced discrimination related to disability (APPR = 2.77 [2.39–3.20], *p* < .001); twice as likely to have faced discrimination related to age (APPR = 2.06 [1.87–2.28], *p* < .001), gender (APPR = 2.01 [1.84–2.19], *p* < .001) and “other” reasons (APPR = 2.00 [1.91–2.10], *p* < .001); just under twice as likely to have faced discrimination related to religion or beliefs (APPR = 1.94 [1.75–2.14], *p* < .001), ethnicity (APPR = 1.88 [1.80–1.96], *p* < .001), and sexual orientation (APPR = 1.83 [1.59–2.10], *p* < .001). All results are adjusted for between-group differences in age.

### Differences in Exposure Associated With Specific Functional Impairments

All functional impairments associated with disability were associated in the age-adjusted models with increased risk of exposure to violence and discrimination, perceived lack of safety and increased risk of IPV (Table 5). Risk of exposure to violence did not significantly vary across different types of functional impairments. In contrast, significantly higher rates of exposure to discrimination were reported by women with impairments in communicating, self-care and remembering than women with visual impairments, and among women with impairments in communicating than among women with impairments in seeing, hearing, walking, and remembering. Significantly higher rates of exposure to unsafe environments were reported by women with impairments in remembering than women with hearing impairments. Significantly higher risk of IPV was evident among women with impairments in remembering or communicating than among women with impairments in walking, and by women with impairments in communicating than among women with impairments in self-care.

**Table 5.** Association between Specific Functional Impairments and Risk of Exposure to Violence, Discrimination, Higher Risk of IPV and Perceptions of Lack of Safety in LMICs.

Functional Limitation	Violence	Discrimination	Higher Risk of IPV	Concern About Safety
Seeing	1.99*** (1.75–2.50)	1.73*** (1.65–1.82)	1.49*** (1.26–1.76)	1.27*** (1.22–1.33)
Hearing	1.76*** (1.54–2.02)	1.87*** (1.75–2.00)	1.48*** (1.32–1.66)	1.19*** (1.13–1.24)
Walking	1.88*** (1.73–2.04)	1.90*** (1.80–2.02)	1.21*** (1.10–1.33)	1.30*** (1.24–1.36)
Remembering	1.89*** (1.72–2.09)	1.97*** (1.87–2.07)	1.57*** (1.40–1.75)	1.34*** (1.27–1.42)
Self-care	1.71*** (1.49–1.97)	2.07*** (1.83–2.33)	1.24** (1.09–1.41)	1.20*** (1.13–1.29)
Communicating	1.80** (1.19–2.74)	2.33*** (2.11–2.59)	1.63*** (1.48–1.81)	1.22*** (1.17–1.28)

*Note*. Kosovo and North Macedonia excluded from violence analysis as data not collected. North Macedonia excluded from safety analysis as data not collected. Zimbabwe and Georgia excluded from IPV acceptability analysis as data not collected. IPV = intimate partner violence; LMIC = low- and middle-income countries.

** *p* < .01. ***p < .001.

### Predictors of Exposure Among Women with Disabilities

Significant within-country predictors of exposure (Table 6) were: lower household wealth (for discrimination, risk of IPV and increased risk of concern about safety); younger age (for discrimination and increased risk of concern about safety); lower education (for risk of IPV), and urban location (for violence and increased risk of concern about safety). Analyses stratified by country economic classification group indicated some marked differences (Supplemental Table 1). For violence, the overall effect of urban location was much stronger in upper-middle and lower-middle income countries and was not significant in low-income countries. However, higher level of education was a significant risk factor for exposure to violence among women with disabilities in low-income countries. For discrimination, the overall effect of household wealth was much stronger in upper-middle and lower-middle income countries and was not significant in low-income countries. For risk if IPV, the overall effects were stronger in upper-middle and lower-middle income countries and were not significant in low-income countries. For increased risk of concern about safety, the association with level of education was not significant in upper-middle or lower-middle income countries, while the effect of urban location and lower household wealth was not significant in lower-income countries.

**Table 6.** Within Country Predictors of Risk of Violence, Discrimination, Risk of IPV, and Low Safety among Women with Disabilities.

	Violence (27 Countries, *n* = 16,383)	Discrimination (29 Countries, *n* = 16,995)	Higher Risk of IPV (27 Countries, *n* = 15,975)	Concern about Safety (28 Countries, *n* = 16,906)
Age				
18–19	1.00 (reference)	1.00 (reference)	1.00 (reference)	1.00 (reference)
20–24	1.16 (0.83–1.63)	0.99 (0.84–1.14)	1.03 (0.92–1.14)	1.01 (0.91–1.12)
25–29	0.97 (0.63–1.51)	0.95 (0.83–1.09)	0.97 (0.86–1.08)	1.01 (0.91–1.13)
30–34	1.07 (0.67–1.73)	0.94 (0.82–1.08)	0.95 (0.85–1.06)	0.96 (0.86–1.07)
35–39	1.03 (0.66–1.59)	0.90 (0.78–1.03)	0.94 (0.83–1.07)	0.93 (0.84–1.03)
40–44	0.77 (0.48–1.25)	0.84* (0.73–0.96)	0.98 (0.88–1.09)	0.91 (0.82–1.01)
45–49	0.68 (0.41–1.12)	0.75*** (0.66–0.86)	0.91 (0.83–1.00)	0.86** (0.78–0.95)
Household wealth				
Quintile 1 (poorest)	1.15 (0.79–1.66)	1.25*** (1.11–1.40)	1.23* (1.01–1.50)	1.15** (1.06–1.25)
Quintile 2	0.92 (0.64–1.33)	1.18** (1.05–1.32)	1.15 (0.96–1.38)	1.11* (1.02–1.20)
Quintile 3	0.94 (0.68–1.30)	1.15* (1.02–1.28)	1.10 (0.97–1.25)	1.10* (1.02–1.19)
Quintile 4	0.94 (0.75–1.18)	1.11* (1.00–1.24)	1.07 (0.96–1.20)	1.08* (1.00–1.17)
Quintile 5 (wealthiest)	1.00 (reference)	1.00 (reference)	1.00 (reference)	1.00 (reference)
Education				
None/pre-primary	0.89 (0.73–1.09)	1.01 (0.92–1.11)	1.20* (1.04–1.39)	0.92* (0.86–0.99)
Primary	1.01 (0.87–1.19)	0.99 (0.92–1.07)	1.30*** (1.14–1.50)	0.99 (0.93–1.04)
Secondary or higher	1.00 (reference)	1.00 (reference)	1.00 (reference)	1.00 (reference)
Location				
Rural	1.00 (reference)	1.00 (reference)	1.00 (reference)	1.00 (reference)
Urban	1.28* (1.04–1.59)	1.07 (1.00–1.15)	0.89 (0.79–1.01)	1.10*** (1.05–1.16)

*Note*. Belarus excluded from analyses as urban/rural data not collected. IPV = intimate partner violence.

* *p* < .05. **p < 0.01. ***p < .001.

### Association With Country Characteristics

Higher national wealth (pcGNI) was significantly associated with: (1) an overall lower prevalence of risk of IPV (*r* = −.57, *p* < .01) and exposure to discrimination (*r* = −.45, *p* < .05); and (2) lower prevalence of exposure of women with disabilities to discrimination (*r* = −.46, *p* < .05), and risk of IPV (*r* = −.55, *p* < .01). It was also significantly associated with lower absolute inequalities in exposure to discrimination (*r* = −.42, *p* < .05). There were non-significant but moderate effect sizes indicating that higher national wealth was associated with a lower overall prevalence of exposure to violence (*r* = −.35, *p* = .07) and feeling unsafe (*r* = −.30, *p* = .12), higher relative inequalities in risk of exposure to discrimination (*r* = +.35, *p* = .07), risk of IPV (*r* = +.35, *p* = .07) and feeling unsafe (*r* = +.30, *p* = .12), lower absolute inequalities in exposure to violence (*r* = −.32, *p* = .10) and higher absolute inequalities in feeling unsafe (*r* = −.32, *p* = .10).

Higher human development potential (HDI) was significantly associated with: (1) an overall lower prevalence of risk of IPV (*r* = +.70, *p* < .001) and exposure to discrimination (*r* = −.56, *p* < .01), violence (*r* = −.52, *p* < .01) and feeling unsafe (*r* = −.41, *p* < .05); (2) lower prevalence of exposure of women with disabilities to discrimination (*r* = −.61, *p* < .001) and risk of IPV (*r* = −.66, *p* < .001); and (3) lower prevalence of exposure of women with disabilities to feeling unsafe (*r* = −.35, *p* = .07), although not significantly. It was also significantly associated with higher relative inequalities in risk of IPV (*r* = +.61, *p* < .001), exposure to discrimination (*r* = +.45, *p* < .05) and feeling unsafe (*r* = +.39, *p* < .05) and lower absolute inequalities in exposure to discrimination (*r* = −.55, *p* < .01). There were non-significant but moderate effect sizes indicating that higher human development potential was associated with higher relative inequalities in risk of exposure to violence (*r* = +.33, *p* = .10), higher absolute inequalities in feeling unsafe (*r* = +.38, *p* = .05) and lower absolute inequalities in exposure to violence (*r* = −.31, *p* = .11).

## Discussion

Our analyses of the circumstances of nationally representative samples involving a total of 320,426 women aged 18 to 49 years from 29 LMICs indicated that: (1) women with disabilities were significantly more likely than women without disabilities to be exposed to violence and discrimination in the past year, to feel unsafe either in their home or in the local neighborhood and to be at higher risk of exposure to IPV; and (2) risk of exposure varied by country characteristics (national wealth and human development potential) and within-country demographics (e.g., household wealth, level of education, urban/rural location). These results must be of concern on two counts. First, they attest to the ongoing violation of the human rights of women with disabilities. Second, they point to increased exposure among women with disabilities to several well-documented social determinants of poorer health.

The results add to existing knowledge in three important ways. First, this is, to our knowledge, the first study to report estimates for the exposure of women with and without disabilities to violence and discrimination over the previous year and exposure to unsafe environments and risk of IPV using nationally representative samples in a range of LMICs. As noted previously, it would clearly be inappropriate to assume that the findings from high-income countries, such as the USA and the UK, would apply to the situation of the majority of the world’s population who live in LMICs. Indeed, our results clearly indicate that within-country risk factors associated with exposure vary markedly by country economic classification group (e.g., strong effects associated with household wealth in high- and middle-income countries do not generalize to low-income countries). Interestingly, a similar effect (weaker association with household assets in poorer countries) has been reported in relation to the prevalence of child disability. One possible reason for this variation is that the MICS household wealth index (typically considered a proxy measure for consumption) may be less valid in low-income countries than in middle-income countries (Howe et al., 2009, 2012; Poirier et al., 2020).

Second, this paper is, to our knowledge, the first paper to report on the breadth of discrimination to which women with disabilities are exposed. Consistent with reports from high-income countries (e.g., Emerson et al., 2021), women with disabilities, in addition to being at increased risk of disability-related discrimination, were also at increased risk of exposure to every other type of discrimination investigated.

Third, our results include analyses of risk factors associated with exposure that may have relevance to the development and implementation of interventions in LMICs to reduce exposure to and/or support the victims of exposure to violence and discrimination. As noted above, these risk factors do show marked variation with national wealth and human development potential. They also show marked variation by measures of exposure.

The main strengths of the study are the use of robust nationally representative samples of women aged 18 to 49 years from 21% of the world’s LMICs with high response rates generated by a UN-driven ongoing survey program. The main weaknesses of the study: (1) the limited nature of the data collected on violence (e.g., no data were collected on sexual violence, actual exposure to IPV, or financial or emotional abuse); (2) the use of the WGSS to identify women with disabilities; and (3) the cross-sectional nature of the data.

The first issue will underestimate exposure to violence. While the failure to collect information on actual exposure to intimate partner violence is of concern, numerous population-based studies in LMICs have reported that women’s attitudes regarding the justifiability of partner to wife violence are predictive of victimization (Abramsky et al., 2011; Cools & Kotsadam, 2017; Heise & Fulu, 2014). As such, our finding that women with disabilities were 30% more likely than women without disabilities to report that partner to wife violence was justifiable in certain circumstances is indicative of potentially higher rates of intimate partner violence victimization among women with disabilities.

The second issue will underestimate the prevalence of disability by its failure to identify women whose disability may be associated with functional impairments not included in the WGSS, for example, women with mental-health-related functional limitations (Bourke et al., 2021; Office for National Statistics, 2019; Sabariego et al., 2015). As such, this may lead to an underestimation of the strength of association between disability and violence and discrimination. Evidence suggests that this may be particularly evident among people with disability associated with mental health impairments (Hughes et al., 2012).

The third issue is methodological: that is, that it is not possible to draw conclusions from cross-sectional data about causation. However, associations bring knowledge further in understanding the situation for women with disabilities in LIMCs compared to the situation of their non-disabled peers and do so in such a way that the effect of specific contexts, for example, household wealth, functional impairments can be appreciated/understood.

The clear implication of these results is the need for all approaches to the prevention of violence or discrimination or support of victims of violence or discrimination in LMICs to make “reasonable accommodations” to their services to ensure that interventions are accessible and effective for the 25% of women victims of violence or discrimination who have a disability. Such accommodations will need to ensure that women with disabilities are as likely as other women to seek help, to participate in intervention programs, and benefit from intervention. Specific attention will need to be given to the situation of women with more severe disabilities whose opportunities to seek help and participate may be restricted by their capacity to, or fear of “speaking out” or denied by the coercive actions of partners or other family members. Otherwise, they are again at risk of being “left behind.” The approach of implementing effective “reasonable accommodations” is fully consistent with the use of “proportionate universalism” to address health inequities (Carey et al., 2015). In addition, the findings that risk of exposure varied by country characteristics (national wealth and human development potential) and within-country demographics (e.g., household wealth, level of education, urban/rural location) suggests a range of possible approaches to systemic preventative interventions including improving access to and the quality of education received by women (including those with disabilities), reducing poverty and reducing income inequalities.

Finally, the use of data from a UN-driven ongoing survey program opens up the possibility of using forthcoming Round 7 MICS data to monitor progress over time in reducing the exposure of women with disabilities to discrimination and violence.

## Supplemental Material

sj-docx-1-jiv-10.1177_08862605221141868 – Supplemental material for Exposure of Women With and Without Disabilities to Violence and Discrimination: Evidence from Cross-sectional National Surveys in 29 Middle- and Low-Income Countries Click here for additional data file. Supplemental material, sj-docx-1-jiv-10.1177_08862605221141868 for Exposure of Women With and Without Disabilities to Violence and Discrimination: Evidence from Cross-sectional National Surveys in 29 Middle- and Low-Income Countries by Eric Emerson and Gwynnyth Llewellyn in Journal of Interpersonal Violence
