# Supplementary material for: Exposure of Women With and Without Disabilities to Violence and Discrimination: Evidence from Cross-sectional National Surveys in 29 Middle- and Low-Income Countries
Source: J Interpers Violence. 2022 Dec 21;38(11-12):7215–41. doi: 10.1177/08862605221141868 (PMC10170557; doi:10.1177/08862605221141868)
Supplement: sj-docx-1-jiv-10.1177_08862605221141868 – Supplemental material for Exposure of Women With and Without Disabilities to Violence and Discrimination: Evidence from Cross-sectional National Surveys in 29 Middle- and Low-Income Countries [file sj-docx-1-jiv-10.1177_08862605221141868.docx]

Supplementary Table 1: Within country predictors of risk of violence, discrimination, risk of IPV and low safety among women with disabilities stratified by country economic classification group

|  | *Violence* | *Discrimination* | *Higher risk of IPV* | *Concern about safety* |
| --- | --- | --- | --- | --- |
| Upper-middle Income | (n=4,325) | (n=4,996) | (n=4,379) | (n=4,822) |
| Age |  |  |  |  |
| 18-19 | 1.00 (reference) | 1.00 (reference) | 1.00 (reference) | 1.00 (reference) |
| 20-24 | 1.90 (0.94-3.86) | 0.96 (0.69-1.33) | 0.79 (0.62-1.01) | 0.98 (0.79-1.22) |
| 25-29 | 1.58 (0.71-3.50) | 0.97 (0.71-1.33) | 1.01 (0.81-1.26) | 1.03 (0.83-1.27) |
| 30-34 | 1.58 (0.85-2.92) | 0.86 (0.63-1.19) | 0.99 (0.81-1.23) | 0.88 (0.71-1.10) |
| 35-39 | 1.63 (0.72-3.68) | 0.83 (0.61-1.14) | 0.79 (0.58-1.06) | 0.86 (0.69-1.05) |
| 40-44 | 1.30 (0.61-2.79) | 0.71* (0.52-0.97) | 0.97 (0.82-1.14) | 0.88 (0.72-1.08) |
| 45-49 | 0.84 (0.41-1.75) | 0.66** (0.49-0.89) | 0.84* (0.72-0.98) | 0.76** (0.62-0.93) |
| Household wealth |  |  |  |  |
| Quintile 1 (poorest) | 1.01 (0.56-1.81) | 1.47** (1.15-1.87) | 1.47*** (1.20-1.79) | 1.31** (1.12-1.53) |
| Quintile 2 | 0.64 (0.33-1.23) | 1.34* (1.05-1.72) | 1.47*** (1.23-1.75) | 1.26* (1.08-1.47) |
| Quintile 3 | 0.62* (0.41-0.96) | 1.26 (0.97-1.62) | 1.33*** (1.18-1.50) | 1.25* (1.07-1.46) |
| Quintile 4 | 0.59* (0.38-0.91) | 1.23 (0.94-1.59) | 1.04 (0.95-1.14) | 1.23* (1.04-1.44) |
| Quintile 5 (wealthiest) | 1.00 (reference) | 1.00 (reference) | 1.00 (reference) | 1.00 (reference) |
| Education |  |  |  |  |
| None/pre-primary | 0.63 (0.33-1.18) | 1.07 (0.86-1.33) | 1.45* (1.02-2.05) | 1.00 (0.87-1.15) |
| Primary | 1.11 (0.84-1.47) | 1.00 (0.86-1.16) | 1.67*** (1.33-2.10) | 1.05 (0.95-1.15) |
| Secondary or higher | 1.00 (reference) | 1.00 (reference) | 1.00 (reference) | 1.00 (reference) |
| Location |  |  |  |  |
| Rural | 1.00 (reference) | 1.00 (reference) | 1.00 (reference) | 1.00 (reference) |
| Urban | 1.76** (1.17-2.65) | 1.03 (0.90-1.18) | 0.78* (0.65-0.93) | 1.09 (0.99-1.19) |

|  |  |  |  |  |
| --- | --- | --- | --- | --- |
| Lower-middle Income | (n=6,553) | (n=6,530) | (n=6,091) | (n=6,562) |
| Age |  |  |  |  |
| 18-19 | 1.00 (reference) | 1.00 (reference) | 1.00 (reference) | 1.00 (reference) |
| 20-24 | 0.90 (0.59-1.37) | 0.85 (0.65-1.11) | 0.93 (0.64-1.33) | 1.03 (0.84-1.25) |
| 25-29 | 0.62 (0.31-1.24) | 0.77* (0.59-1.00) | 0.82 (0.52-1.28) | 0.93 (0.76-1.13) |
| 30-34 | 0.75 (0.34-1.63) | 0.78 (0.61-1.01) | 0.82 (0.54-1.24) | 0.95 (0.79-1.15) |
| 35-39 | 0.57 (0.32-1.01) | 0.77* (0.60-0.98) | 0.99 (0.71-1.37) | 0.86 (0.71-1.03) |
| 40-44 | 0.41** (0.22-0.77) | 0.68** (0.53-0.87) | 0.89 (0.63-1.25) | 0.83* (0.69-0.99) |
| 45-49 | 0.36** (0.18-0.72) | 0.63*** (0.49-0.80) | 0.85 (0.63-1.16) | 0.79* (0.66-0.95) |
| Household wealth |  |  |  |  |
| Quintile 1 (poorest) | 1.82 (0.83-4.00) | 1.49*** (1.21-1.84) | 2.13*** (1.77-2.58) | 1.17* (1.01-1.36) |
| Quintile 2 | 1.37 (0.69-2.70) | 1.42** (1.16-1.74) | 1.83*** (1.42-2.35) | 1.13* (0.98-1.30) |
| Quintile 3 | 1.39 (0.72-2.68) | 1.29* (1.05-1.57) | 1.56*** (1.28-1.90) | 1.10 (0.96-1.26) |
| Quintile 4 | 1.34 (0.88-2.02) | 1.22* (1.00-1.48) | 1.49*** (1.24-1.80) | 1.05 (0.92-1.20) |
| Quintile 5 (wealthiest) | 1.00 (reference) | 1.00 (reference) | 1.00 (reference) | 1.00 (reference) |
| Education |  |  |  |  |
| None/pre-primary | 1.18 (0.92-1.52) | 1.14 (0.95-1.37) | 1.43* (1.02-1.99) | 0.94 (0.82-1.08) |
| Primary | 1.15 (0.94-1.41) | 1.02 (0.90-1.16) | 1.29** (1.06-1.56) | 0.94 (0.86-1.03) |
| Secondary or higher | 1.00 (reference) | 1.00 (reference) | 1.00 (reference) | 1.00 (reference) |
| Location |  |  |  |  |
| Rural | 1.00 (reference) | 1.00 (reference) | 1.00 (reference) | 1.00 (reference) |
| Urban | 1.49*** (1.26-1.76) | 1.23** (1.09-1.40) | 0.93 (0.81-1.07) | 1.13** (1.03-1.24) |
|  |  |  |  |  |
| Low-Income | (n=5,505) | (n=5,469) | (n=5,505) | (n=5.522) |
| Age |  |  |  |  |
| 18-19 | 1.00 (reference) | 1.00 (reference) | 1.00 (reference) | 1.00 (reference) |
| 20-24 | 1.15 (0.75-1.75) | 1.09 (0.90-1.31) | 1.10* (1/01-1.20) | 1.00 (0.86-1.18) |
| 25-29 | 1.12 (0.78-1.62) | 1.03 (0.85-1.25) | 1.01 (0.90-1.14) | 1.05 (0.90-1.23) |
| 30-34 | 1.17 (0.69-1.98) | 1.05 (0.87-1.28) | 0.99 (0.87-1.13) | 0.98 (0.84-1.15) |
| 35-39 | 1.42 (0.92-2.20) | 0.98 (0.81-1.19) | 0.97 (0.83-1.13) | 1.02 (0.87-1.19) |
| 40-44 | 1.04 (0.65-1.66) | 1.00 (0.83-1.21) | 0.99 (0.84-1.17) | 0.99 (0.85-1.15) |
| 45-49 | 1.13 (0.64-2.03) | 0.85 (0.71-1.04) | 0.93 (0.81-1.05) | 1.01 (0.86-1.17) |
| Household wealth |  |  |  |  |
| Quintile 1 (poorest) | 0.93 (0.63-1.38) | 1.08 (0.91-1.28) | 1.01 (0.84-1.22) | 1.08 (0.94-1.24) |
| Quintile 2 | 0.88 (0.55-1.42) | 1.02 (0.85-1.22) | 0.99 (0.87-1.11) | 1.03 (0.89-1.19) |
| Quintile 3 | 0.88 (0.51-1.53) | 1.05 (0.89-1.25) | 1.02 (0.91-1.14) | 1.05 (0.91-1.20) |
| Quintile 4 | 0.93 (0.73-1.18) | 1.03 (0.88-1.20) | 1.03 (0.98-1.09) | 1.05 (0.93-1.19) |
| Quintile 5 (wealthiest) | 1.00 (reference) | 1.00 (reference) | 1.00 (reference) | 1.00 (reference) |
| Education |  |  |  |  |
| None/pre-primary | 0.68*** (0.59-0.79) | 0.91 (0.80-1.05) | 1.04 (0.95-1.13) | 0.88* (0.78-0.99) |
| Primary | 0.79* (0.64-1.97) | 0.93 (0.83-1.06) | 1.12 (0.98-1.30) | 0.99 (0.90-1.10) |
| Secondary or higher | 1.00 (reference) | 1.00 (reference) | 1.00 (reference) | 1.00 (reference) |
| Location |  |  |  |  |
| Rural | 1.00 (reference) | 1.00 (reference) | 1.00 (reference) | 1.00 (reference) |
| Urban | 1.01 (0.65-1.58) | 0.97 (0.86-1.10) | 0.93 (0.86-1.02) | 1.08 (0.98-1.19) |

Note: Belarus excluded from analyses as urban/rural data not collected. * p<0.05, ** p<0.01, *** p<0.001
